# Case Report: Coexisting cold agglutinin disease and acquired hemophilia A: a rituximab-responsive dual autoimmune disorder

**DOI:** 10.3389/fmed.2025.1673125

**Published:** 2025-10-17

**Authors:** Congcong Sun, Jingyi Yu, Jing Sun, Xiaofei Jia, Wenxin Ma, Saran Feng, Yan Wang, Ruirong Xu

**Affiliations:** ^1^Post-Doctoral Mobile Research Station, College of Integrated Traditional Chinese and Western Medicine, Shandong University of Traditional Chinese Medicine, Jinan, China; ^2^Department of Hematology, The First Affiliated Hospital of Shandong First Medical University & Shandong Provincial Qianfoshan Hospital, Jinan, China; ^3^Shandong Center for Food and Drug Evaluation and Inspection, Jinan, Shandong, China; ^4^Product R&D, Inspur Computer Technology Co., Ltd., Jinan, China; ^5^Department of Hematology, Affiliated Hospital of Shandong University of Traditional Chinese Medicine, Jinan, China; ^6^Institute of Hematology, Shandong University of Traditional Chinese Medicine, Jinan, Shandong, China; ^7^Key Laboratory of Integrated Traditional Chinese and Western Medicine for Hematology, Health Commission of Shandong Province, Shandong, China

**Keywords:** cold agglutinin disease, acquired hemophilia A, autoimmune hemolytic anemia, rituximab, factor VIII inhibitor

## Abstract

This report describes the first documented case of concurrent cold agglutinin disease (CAD) and acquired hemophilia A (AHA) in a 53-year-old male presenting with recurrent hematuria, hematemesis, and cold-induced acrocyanosis. Diagnostic findings included severe anemia with hemoglobin of 61 g/L, markedly prolonged activated partial thromboplastin time (aPTT, 88.6 s), critically reduced factor VIII activity (1.4%), a factor VIII inhibitor titer of 3.6 Bethesda units, and an elevated cold agglutinin titer of 1:320. Initial immunosuppression with corticosteroids and cyclophosphamide failed to improve either the coagulopathy or hemolytic anemia, consistent with the recognized poor response of CAD to steroid therapy. Clinical deterioration occurred during steroid tapering, complicated by hospital-acquired pneumonia. Administration of rituximab (375 mg/m^2^ weekly for 4 weeks) resulted in simultaneous resolution of both autoimmune processes, with normalization of coagulation parameters and significant improvement in hemoglobin levels. This outcome aligns with established evidence supporting B-cell targeted therapy for autoimmune hematologic disorders. The case highlights the diagnostic challenges posed by overlapping autoimmune hematologic conditions and demonstrates the therapeutic potential of rituximab in simultaneously addressing both coagulation and hemolytic pathologies. It further underscores the importance of targeted treatment strategies that minimize infection risks associated with broad immunosuppression. This unique presentation advances our understanding of shared autoimmune mechanisms in hematologic disease and supports the use of early B-cell-directed therapy in complex autoimmune hematologic conditions.

## 1 Introduction

Autoimmune hemolytic anemia (AIHA) is a hematological disorder characterized by the premature destruction of red blood cells (RBCs) mediated by autoantibodies. AIHA is classified into warm AIHA and cold AIHA based on the thermal reactivity of the autoantibodies (1). Cold agglutinin disease (CAD), a distinct form of cold AIHA, is characterized by hemolysis that is primarily complement-mediated and exacerbated upon exposure to cold temperatures (2). Another, rarer form is paroxysmal cold hemoglobinuria (PCH). Clinically, CAD patients may present with acrocyanosis and hemoglobinuria upon cold exposure, alongside fatigue and signs of anemia. The diagnosis is supported by laboratory findings including a positive direct antiglobulin test (DAT), which is typically positive for complement C3d but negative for IgG, elevated lactate dehydrogenase (LDH), and increased indirect bilirubin levels (3). The presence of cold agglutinins, typically monoclonal IgM antibodies with a titer ≥1:64 at 4 °C, is a serological hallmark (4).

AHA is a rare bleeding disorder caused by autoantibodies (inhibitors) against coagulation factor VIII. It is often associated with autoimmune conditions, malignancies, pregnancy, or it may be idiopathic (5). The clinical presentation includes spontaneous bleeding into the skin, muscles, and soft tissues, which can be severe and life-threatening. The diagnosis is confirmed by an isolated prolonged activated partial thromboplastin time (aPTT) that does not correct in mixing studies, severely reduced factor VIII activity, and the detection of a factor VIII inhibitor via assays such as the Bethesda assay (6).

The co-occurrence of CAD and AHA in a single patient is exceedingly rare. This case report describes a patient who simultaneously presented with both autoimmune disorders, a scenario with significant diagnostic and therapeutic implications.

## 2 Case report

A 53-year-old male was admitted with a 4-year history of recurrent tea-colored urine (suggestive of hemoglobinuria) and a 1-day episode of epistaxis. His medical history was significant for episodes of hemoglobinuria and acrocyanosis (e.g., in fingers and ears) triggered by cold exposure, which resolved upon warming. One month prior to admission, he experienced an episode of hematemesis, accompanied by dark brown urine, chest tightness, and fatigue. Initial laboratory investigations at an outside clinic revealed leukocytosis (11.18 × 10^9^/L), severe anemia (hemoglobin: 74 g/L), and normal platelet count (388 × 10^9^/L). Coagulation studies showed a prolonged aPTT (49.2 s). Biochemical markers indicated hemolysis, with elevated total bilirubin (89.6 μmol/L) and unconjugated bilirubin (84.4 μmol/L). Factor VIII activity was severely reduced to 22.8% (normal range: 50–150%), and a qualitative factor VIII inhibitor was detected, confirming a concomitant diagnosis of acquired hemophilia A (AHA). Von Willebrand factor antigen (VWF:Ag) was elevated at 238.5%. A high-sensitivity paroxysmal nocturnal hemoglobinuria (PNH) clone test was negative. Upper endoscopy revealed submucosal bleeding in the esophagus. The patient received the following interventions: intravenous immunoglobulin (IVIG) therapy for immunomodulation, hemostatic agents (e.g., tranexamic acid) to control bleeding, broad-spectrum antibiotics for infection prophylaxis and proton pump inhibitors for gastric mucosal protection. Following this intervention, his overt gastrointestinal bleeding stabilized. However, his coagulopathy and hemolytic anemia persisted, as evidenced by a persistently prolonged aPTT and worsening anemia.

**Figure 1:**
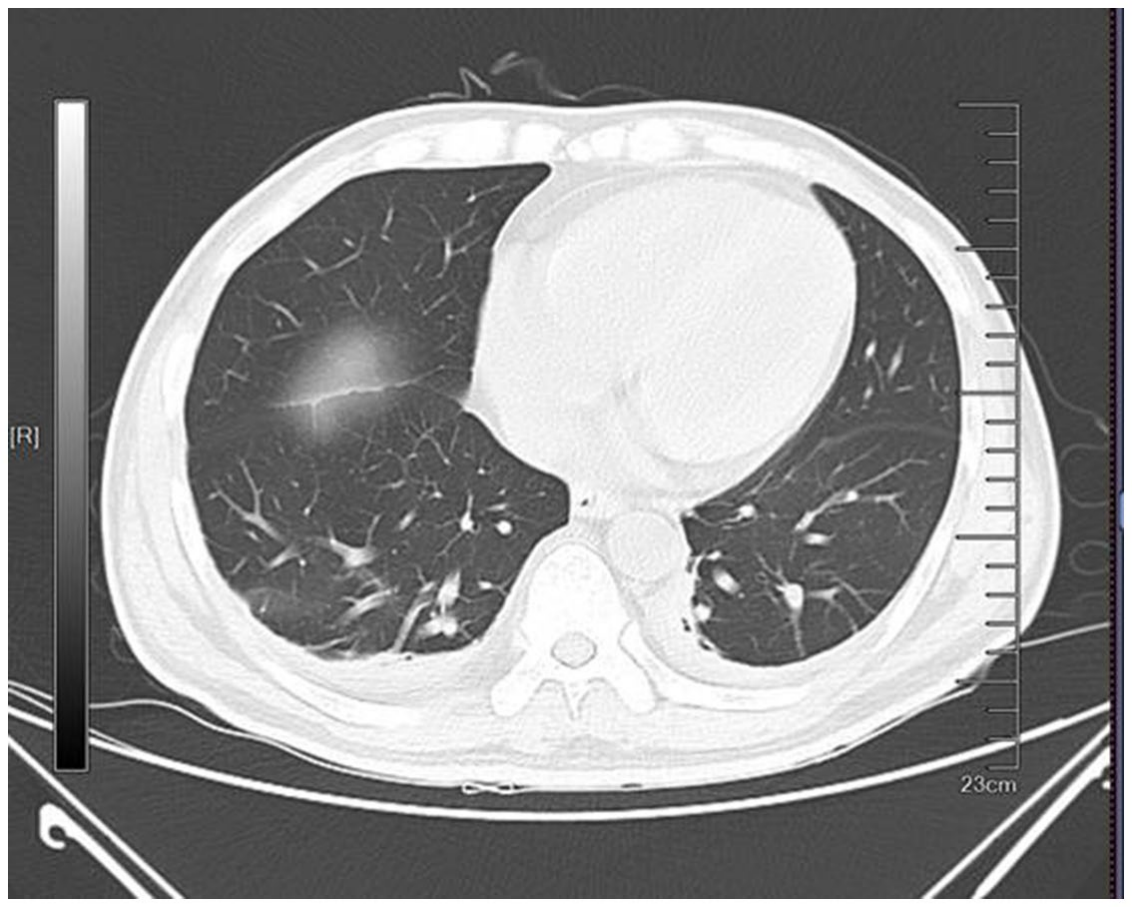
CT image of the patient’s chest. The CT showed bilateral pulmonary exudative changes and bilateral pleural effusion.

**Figure 2:**
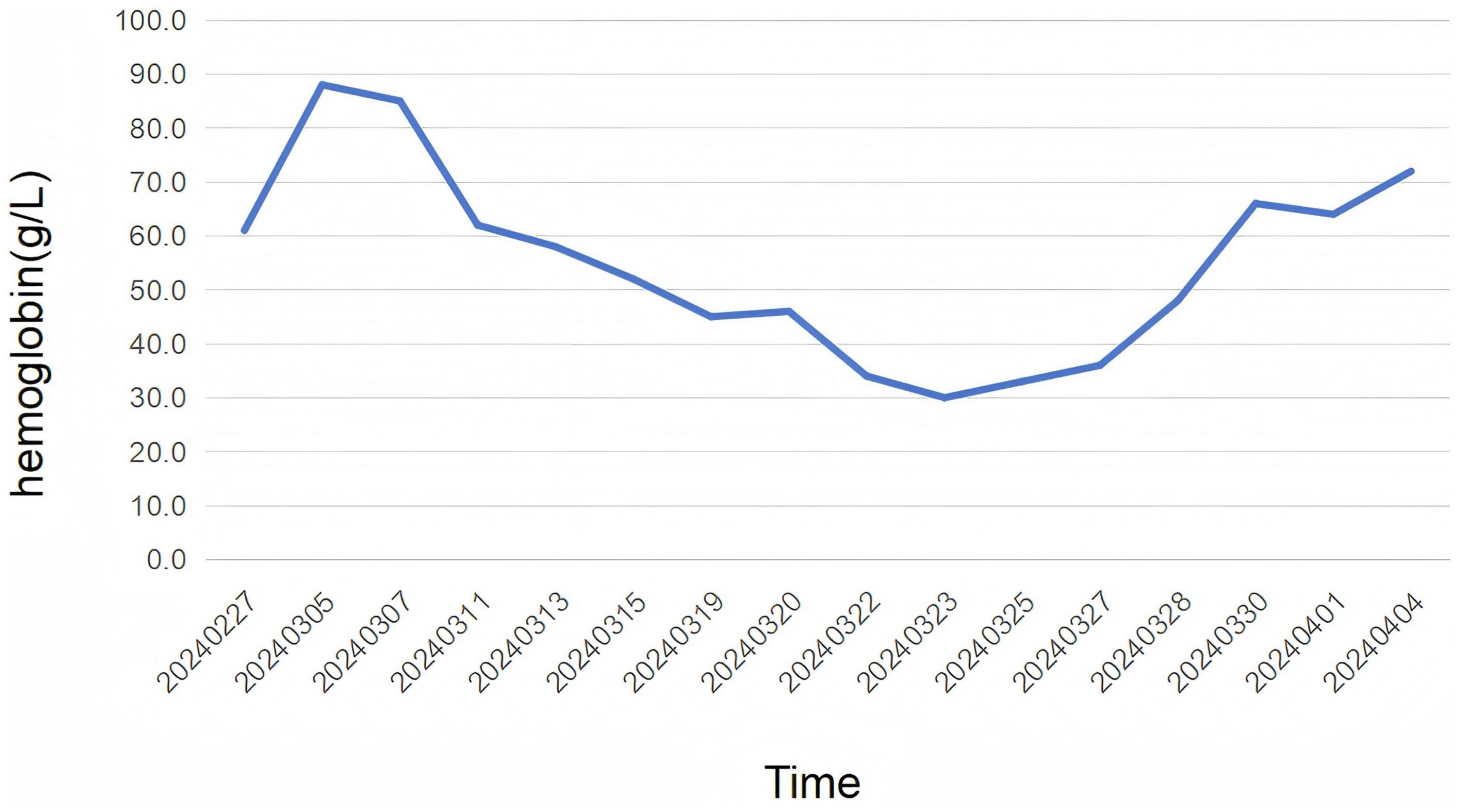
The figure illustrates the levels of hemoglobin over time. The *x*-axis represents the dates, spanning from 02/27/2024–04/04/2024, while the *y*-axis represents the hemoglobin levels in g/L. The patient received prednisone (1 mg/kg) on 02/27/2024, indicated by the first data point. The graph shows an trend of changes in hemoglobin. The patient received methylprednisolone of 200 mg on 11/03/2024, as the second data point. The last data points, from 16/03/2024–04/04/2024, the patient received rituximab (375 mg/m^2^) for 4 weeks.

**Figure 3:**
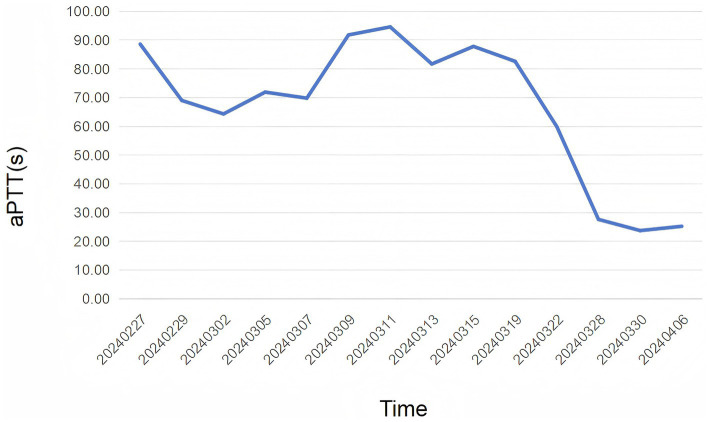
The figure illustrates the levels of aPTT over time. The *x*-axis represents the dates, spanning from 02/27/2024–04/06/2024, while the *y*-axis represents the aPTT levels in seconds. The patient received prednisone (1 mg/kg) on 02/27/2024, indicated by the first data point. The graph shows an trend of changes in aPTT. The patient received methylprednisolone of 200 mg on 11/03/2024, as the second data point. The last data points, from 16/03/2024–04/04/2024, the patient received rituximab (375 mg/m^2^) for 4 weeks.

Physical examination revealed pallor of the conjunctivae and palmar creases, tachycardia (heart rate: 112 bpm), and active epistaxis. No icteric sclera, splenomegaly, or petechiae were observed. Complete blood count revealed leukocyte counts of 5.94 × 10^9^/L [normal (3.5–9.5) × 10^9^/L], severe anemia with a hemoglobin level of 61.0 g/L [normal 130–175 g/L], and a platelet count of 345 × 10^9^/L [normal (125–350) × 10^9^/L]. The erythrocyte indices showed macrocytosis (MCV 111.9 fL) and a decreased mean corpuscular hemoglobin concentration (MCHC 295 g/L). Peripheral blood smear examination revealed: marked RBC agglutination at room temperature, spherocytes (approximately 15% of RBCs), absence of schistocytes, microspherocytes, or bite cells, polychromasia (indicating reticulocytosis), and leukocyte and platelet morphology within normal limits. A direct antiglobulin test (DAT) was positive, notably for complement C3d, which is characteristic of CAD. The aPTT was significantly prolonged at 88.60 s. Biochemical markers of hemolysis were elevated: total bilirubin was 43.9 μmol/L (with an indirect bilirubin predominance of 29.00 μmol/L), and lactate dehydrogenase was 274 U/L. Coagulation studies confirmed the diagnosis of AHA, with a factor VIII activity level of 1.4% and a factor VIII inhibitor titer of 3.6 Bethesda units (BU). Serological testing demonstrated a high cold agglutinin titer of 1:320, which is diagnostic for CAD. G6PD activity was normal at 12.5 U/g Hb (reference: 7.0–20.5 U/g Hb). ADAMTS13 activity was 78% (reference: ≥67%). The immunofixation was negative. A comprehensive evaluation was undertaken to identify underlying triggers. Serological tests for common autoimmune connective tissue diseases (e.g., antinuclear antibodies, rheumatoid factor) were negative. No evidence of solid malignancy was found on contrast-enhanced CT imaging of the chest, abdomen, and pelvis, or on endoscopic evaluation. Serum protein electrophoresis and immunofixation showed no monoclonal gammopathy, excluding a plasma cell dyscrasia. Serologies for acute hepatitis (A, B, C), HIV, and Epstein–Barr virus were likewise negative. Thus, this case was classified as idiopathic. Diagnostic evaluation of AHA adhered to standardized protocols: blood smear excluded microangiopathic hemolysis, DIC was objectively ruled out by ISTH criteria using serial coagulation profiles, G6PD/PNH testing and ADAMTS13 activity confirmed alternative etiologies were unlikely. The diagnosis of CAD was confirmed based on the presence of chronic hemolytic anemia, characteristic cold-induced acrocyanosis and hemoglobinuria, a positive DAT, and a significantly elevated cold agglutinin titer of 1:320, which is well above the diagnostic threshold of 1:64 at 4 °C, in the absence of underlying lymphoproliferative disorders, infections, or other secondary causes. Concurrently, the diagnosis of AHA was established by the presence of spontaneous bleeding episodes (including epistaxis and historical gastrointestinal bleeding), markedly prolonged activated partial thromboplastin time (aPTT), severely reduced factor VIII activity (1.4%), and the detection of a factor VIII inhibitor at 3.6 Bethesda units, confirming an autoimmune etiology for the coagulopathy. Thus, based on the patient’s clinical manifestations and laboratory findings, a definitive diagnosis of concurrent CAD and AHA was established. The co-occurrence of these two rare autoimmune disorders suggests a shared underlying immune pathophysiology, potentially involving epitope spreading in a genetically susceptible individual. Initial immunosuppressive therapy consisted of corticosteroids (prednisone 1 mg/kg/day) and cyclophosphamide (100 mg/day). However, consistent with literature indicating poor response to steroids in CAD (2, 3), the patient showed minimal improvement. Pulse therapy with methylprednisolone (200 mg daily for 4 days) was subsequently administered, followed by a gradual taper. In addition, prothrombin complex concentrate (PCC) and packed red blood cells (PRBCs) were transfused to improve coagulation function and correct anemia, respectively. During the corticosteroid taper, the patient developed hospital-acquired pneumonia (Figure 1), which progressed to septic shock—a known risk associated with intensive immunosuppression. Concurrently, laboratory parameters worsened, with recurrent prolongation of aPTT and declining hemoglobin levels. Given the inadequate response to initial therapy and the complications encountered, treatment with rituximab (375 mg/m^2^ weekly for 4 weeks) was initiated, reflecting a targeted B-cell depletion strategy supported by evidence in both CAD and AHA (2, 7). Following rituximab administration, the patient’s aPTT normalized, indicating a promising biochemical response. The dynamic changes in hemoglobin and aPTT throughout the clinical course are illustrated in Figures 2, 3.

## 3 Discussion

Existing studies have documented cases of CAD and AHA separately (8–10), but the co-occurrence of these two conditions remains underexplored. This is the first reported case of CAD and AHA occurring simultaneously. The presented case highlights the importance of recognizing overlapping autoimmune disorders, particularly in patients exhibiting atypical clinical presentations. This case is significant not only because it illustrates the rare co-occurrence of these conditions but also because it underscores the complexity involved in the diagnosis and management of such patients.

The prevailing hypotheses suggest that the autoimmune mechanisms driving AIHA could also contribute to the development of inhibitors against factor VIII, especially in patients with underlying autoimmune diseases (11). This notion is reinforced by our case, where the patient presented with a positive Coombs test and significant coagulation abnormalities, indicative of an underlying immune-mediated process. Thus clinicians should maintain a high index of suspicion for concurrent hematological disorders in patients with atypical presentations especially when laboratory findings indicate abnormalities such as prolonged aPTT and positive Coombs tests.

The management of CAD is stratified by disease severity, with pharmacological intervention recommended for symptomatic anemia (hemoglobin ≤10 g/dL), transfusion dependence, or cold-induced circulatory impairment per 2023 International Guidelines (12). The management of CAD focuses on suppressing pathogenic IgM autoantibody production and inhibiting complement-mediated hemolysis. First-line therapy involves rituximab (anti-CD20 monoclonal antibody), achieving 50–60% response rates as monotherapy (13) or 70% when combined with bendamustine (14). Complement-targeted agents like sutimlimab (C1s inhibitor) provide rapid hemoglobin normalization in complement-mediated hemolysis (15), while novel strategies such as bruton tyrosine kinase (BTK) inhibitors (16) or proteasome inhibitor bortezomib (17) show promise in refractory cases. Supportive care includes thermal protection and warmed blood transfusions for critical anemia. Current management of AHA combines immediate hemostasis with immunosuppressive eradication of FVIII inhibitors. The primary agents utilized for the management of hemorrhage comprise recombinant activated factor VII (rFVIIa) and activated prothrombin complex concentrate (aPCC), both of which possess the capability to circumvent the obstruction of the coagulation cascade induced by the FVIII inhibitory autoantibody (11). First-line eradication therapy includes corticosteroids ± cyclophosphamide, achieving complete remission in 48–70% of cases within 1–2 months (18). Emerging strategies integrate rituximab as a steroid-sparing alternative, demonstrating comparable efficacy (77% remission rate) to cyclophosphamide with improved long-term safety profiles (19). For refractory bleeding, subcutaneous emicizumab provides rapid hemostatic support through its bispecific antibody mechanism, though requires thrombotic risk monitoring when combined with aPCC (20). Treatment selection increasingly emphasizes individualized risk stratification, balancing bleeding severity, inhibitor kinetics, and comorbidity profiles (particularly in elderly or malignancy-associated cases), while ongoing research aims to optimize therapeutic sequencing and relapse prevention protocols. In our case, the initial first-line immunosuppression with prednisone and cyclophosphamide aligned with standard AHA protocols but resulted in suboptimal response, highlighting the 20–30% resistance rate to conventional therapies. The subsequent development of severe pneumonia during steroid tapering underscores the infection risks associated with prolonged high-dose immunosuppression, particularly in comorbid patients. The successful use of rituximab (375 mg/m^2^ weekly for 4 weeks) concurrently addressed both pathologies through B-cell suppression—normalizing coagulation parameters (aPTT) while likely mitigating cold agglutinin-mediated hemolysis. The concurrent presentation of CAD and AHA, though rare, highlights a shared underlying etiology of B-cell dysregulation and autoantibody production. The poor response of CAD to corticosteroids, as demonstrated in our case and well-documented in the literature (3, 21), underscores the need for a targeted therapeutic strategy. Rituximab, a B-cell-depleting agent, presents a rational first-line treatment for such cases. Its proven efficacy in CAD and reported utility in AHA stem from its ability to target the common pathogenic mechanism: the autoantibody-producing B-cell clone (2, 7). Therefore, integrating rituximab earlier in the treatment algorithm for patients with concurrent autoimmune cytopenias could mitigate the risks associated with ineffective immunosuppressive therapies and improve overall outcomes. Prothrombin complex provided transient hemostasis but failed to address ongoing bleeding, emphasizing the need for more sustained solutions like emicizumab in refractory cases. Collectively, this case reinforces the necessity for personalized treatment algorithms integrating bleeding severity, autoimmune burden, and infection risk stratification.

In conclusion, this case of simultaneous CAD and AHA not only enriches the current literature but also paves the way for further exploration into the autoimmune underpinnings of these disorders. The intricate interplay between CAD and AHA suggests that future research should focus on elucidating the underlying mechanisms that predispose patients to these dual diagnoses. Moreover, the therapeutic strategies employed in this case underscore the importance of individualized treatment plans, balancing the risks and benefits of immunosuppressive therapies while considering the overall health status of the patient.

## 4 Patient outcome

Following the four-week course of rituximab, the patient was discharged and monitored closely in the outpatient clinic. The aPTT remained normalized, and the FVIII inhibitor became undetectable, indicating complete remission of AHA. The hemoglobin level demonstrated a gradual and sustained improvement, stabilizing between 108–115 g/L, consistent with a partial remission of CAD as defined by standardized criteria. The patient reported a significant resolution of fatigue and no further episodes of bleeding or hemoglobinuria. Oral cyclophosphamide, initiated during the initial hospitalization for broad immunosuppression, was continued at a low dose (100 mg/day) as maintenance therapy to mitigate the risk of relapse. All financial, commercial or other relationships that might be perceived by the academic community as representing a potential conflict of interest must be disclosed.

## Data Availability

The original contributions presented in the study are included in the article/supplementary material, further inquiries can be directed to the corresponding authors.

## References

[1] Hill A Hill QA. Autoimmune hemolytic anemia. Hematology. (2018) 2018:382–9. doi: 10.1182/asheducation-2018.1.382, PMID: 30504336 PMC6246027

[2] Berentsen S. Cold agglutinin disease. Hematology Am Soc Hematol Educ Program. (2016) 2016:226–31. doi: 10.1182/asheducation-2016.1.226, PMID: 27913484 PMC6142439

[3] Gabbard AP Booth GS. Cold agglutinin disease. Clin Hematol Int. (2020) 2:95–100. doi: 10.2991/chi.k.200706.001, PMID: 34595449 PMC8432332

[4] Michel M Crickx E Fattizzo B Barcellini W. Autoimmune haemolytic anaemias. Nat Rev Dis Primers. (2024) 10:82. doi: 10.1038/s41572-024-00566-2, PMID: 39487134

[5] Gertz MA. Cold agglutinin disease and cryoglobulinemia. Clin Lymphoma. (2005) 5:290–3. doi: 10.3816/CLM.2005.n.019, PMID: 15794868

[6] Lévesque H Guillet B d’Oiron R Benhamou Y. Hémophilie acquise: quoi de neuf en 2024? Rev Med Interne. (2024) 45:710–25. doi: 10.1016/j.revmed.2024.06.005, PMID: 39245591

[7] Camou F Viallard JF Pellegrin JL. Intérêt du rituximab dans la maladie des agglutinines froides. Rev Med Interne. (2003) 24:501–4. doi: 10.1016/S0248-8663(03)00139-5, PMID: 12888170

[8] Ayoobkhan FS Padmanabhan DS Mahayni R Riaz S Krishnamoorthy G. Unravelling acquired hemophilia A in an ambiguous clinical picture. Cureus. (2024) 16:e68549. doi: 10.7759/cureus.68549, PMID: 39364497 PMC11449379

[9] Launois A Martin-Toutain I Devaux F Lambert J Longval T Merabet F. Hémophilie A acquise, le traitement par emicizumab peut relayer les agents by-passants: à propos de deux cas et une revue de la littérature. Ann Biol Clin. (2024) 82:294–307. doi: 10.1684/abc.2024.1900 39150152

[10] Zhao H Chen J Ou G. A case report of severe drug-induced immune hemolytic anemia caused by piperacillin. Front Immunol. (2024) 15:1478545. doi: 10.3389/fimmu.2024.1478545, PMID: 39569195 PMC11576437

[11] Coppola A Franchini M Tripodi A Santoro RC Castaman G Marino R. Acquired haemophilia a: Italian consensus recommendations on diagnosis, general management and treatment of bleeding. Blood Transfus. (2022) 20:245–62. doi: 10.2450/2022.0238-21, PMID: 35175184 PMC9068356

[12] Berentsen S Tjønnfjord GE. Diagnosis and treatment of cold agglutinin mediated autoimmune hemolytic anemia. Blood Rev. (2012) 26:107–15. doi: 10.1016/j.blre.2012.01.002, PMID: 22330255

[13] Berentsen S Barcellini W. Autoimmune hemolytic anemias. N Engl J Med. (2021) 385:1407–19. doi: 10.1056/NEJMra2033982, PMID: 34614331

[14] Berentsen S Randen U Oksman M Birgens H Tvedt THA Dalgaard J. Bendamustine plus rituximab for chronic cold agglutinin disease: results of a Nordic prospective multicenter trial. Blood. (2017) 130:537–41. doi: 10.1182/blood-2017-04-778175, PMID: 28533306

[15] Berentsen S Barcellini W D’Sa S Jilma B. Sutimlimab for treatment of cold agglutinin disease: why, how and for whom? Immunotherapy. (2022) 14:1191–204. doi: 10.2217/imt-2022-0085, PMID: 35946351

[16] Jalink M Berentsen S Castillo JJ Treon SP Cruijsen M Fattizzo B. Effect of ibrutinib treatment on hemolytic anemia and acrocyanosis in cold agglutinin disease/cold agglutinin syndrome. Blood. (2021) 138:2002–5. doi: 10.1182/blood.2021012039, PMID: 34293088

[17] Rossi G Gramegna D Paoloni F Fattizzo B Binda F D’Adda M. Short course of bortezomib in anemic patients with relapsed cold agglutinin disease: a phase 2 prospective GIMEMA study. Blood. (2018) 132:547–50. doi: 10.1182/blood-2018-03-835413 29898955

[18] Tiede A Klamroth R Scharf RE Trappe RU Holstein K Huth-Kühne A. Prognostic factors for remission of and survival in acquired hemophilia A (AHA): results from the GTH-AH 01/2010 study. Blood. (2015) 125:1091–7. doi: 10.1182/blood-2014-07-587089, PMID: 25525118 PMC4326770

[19] Franchini M Mannucci PM. Inhibitor eradication with rituximab in haemophilia: where do we stand? Br J Haematol. (2014) 165:600–8. doi: 10.1111/bjh.12829, PMID: 24628543

[20] Knoebl P Thaler J Jilma P Quehenberger P Gleixner K Sperr WR. Emicizumab for the treatment of acquired hemophilia A. Blood. (2021) 137:410–9. doi: 10.1182/blood.2020006315, PMID: 32766881

[21] Berentsen S. Chronic cold agglutinin disease. Tidsskr Nor Laegeforen. (1995) 115:473–5. 7871504

